# Deceleration capacity as a risk predictor in patients presenting to the emergency department with syncope: A prospective exploratory pilot study

**DOI:** 10.1097/MD.0000000000008605

**Published:** 2017-12-08

**Authors:** Martin Duckheim, Katharina Klee, Nina Götz, Paul Helle, Patrick Groga-Bada, Lars Mizera, Meinrad Gawaz, Christine S. Zuern, Christian Eick

**Affiliations:** a Department of Cardiology, Innere Medizin III, Eberhard-Karls-Universität Tübingen, Tübingen; b Department of Internal Medicine, Filderklinik Stuttgart, Stuttgart, Germany.

**Keywords:** cardiac autonomic dysfunction, deceleration capacity, emergency medicine, mortality, risk markers, syncope

## Abstract

Syncope is a common cause for admission to the emergency department (ED). Due to limited clinical resources there is great interest in developing risk stratification tools that allow identifying patients with syncope who are at low risk and can be safely discharged. Deceleration capacity (DC) is a strong risk predictor in postinfarction and heart failure patients. The aim of this study was to evaluate whether DC provides prognostic information in patients presenting to ED with syncope.

We prospectively enrolled 395 patients presenting to the ED due to syncope. Patient's electrocardiogram (ECG) for the calculation of DC was recorded by monitoring devices which were started after admission. Both the modified early warning score (MEWS) and the San Francisco syncope score (SFSS) were determined in every patient. Primary endpoint was mortality after 180 days.

Eight patients (2%) died after 180 days. DC was significantly lower in the group of nonsurvivors as compared with survivors (3.1 ± 2.5 ms vs 6.7 ± 2.4 ms; *P* < .001), whereas the MEWS was comparable in both was comparable in both groups. (2.1 ± 0.8 vs 2.1 ± 1.0; *P* = .84). The SFSS failed at identifying 4 of 8 nonsurvivors (50%) as high risk patients. No patient with a favorable DC (≥7 ms) died (0.0% vs 3.7%; *P* = .01, OR 0.55 (95% CI 0.40–0.76), *P* < .001). In the receiver operating characteristic (ROC) analysis DC yielded an area under the curve of 0.85 (95% CI 0.71–0.98).

Our study demonstrates that DC is a predictor of 180-days-mortality in patients admitted to the ED due to syncope. Syncope patients at low risk can be identified by DC and may be discharged safely.

## 1 Introduction

Due to demographic changes and deficits in ambulatory care emergency department (ED) overcrowding has become a serious problem, triggering both a suboptimal patient care and an increase in mortality.^[1,2]^ One common cause for presentation to ED is syncope—approximately 740,000 annual visits in the United States.^[3]^ Since syncope can be caused by dangerous conditions, many patients are admitted to inpatient care for further investigations despite an initial inconspicuous ED evaluation.^[4]^ However, the majority of these inpatient diagnostics remain inconclusively.^[5]^ Due to optimization of clinical resources there is great interest in developing a risk stratification tool that allows the ED physician to identify reliably and discharge safely patients with syncope who are at low risk. Accordingly, many risk models were built in the last decade, but none of them has been permanently implemented into daily clinical work.^[6–12]^ The “modified early warning score” (MEWS) as one of the conventional risk scores can be easily calculated by nursing staff. Previous studies revealed that the addition of MEWS to clinical judgment indeed increases sensitivity but with the expense of reduced specificity.^[13]^ A further risk score is called the San Francisco syncope score (SFSS), which identifies syncope patients at risk by screening them referring to heart failure, shortness of breath, Electrocardiography (ECG) changes, low hematocrit, and systolic blood pressure.^[9]^ However, an independent validation study showed that 26% of patients with serious outcomes were not identified by SFSS.^[14]^

Therefore, the identification of novel tools that allow for risk stratification and safe discharge management of patients with syncope is of great general interest.

Essential information about the current clinical condition of a patient can be determined by the assessment of the cardiac autonomous nervous system (ANS).^[15]^ The ANS is a neuronal network connecting all organ systems. Any harm of one of these systems leads to an autonomic dysfunction, which can be quantified by autonomic parameters. The strong and independent prognostic value of these markers has been already demonstrated in patients with heart failure, myocardial infarction, and aortic stenosis.^[16–19]^ Recently, we were able to identify deceleration capacity (DC) as one of these cardiac autonomic parameters to be an independent risk predictor in all-comers presenting to the ED, independent of the underlying condition.^[15]^

The aim of this study was to test whether DC provides prognostic information in patients admitted to the ED due to syncope.

## 2 Methods

### 2.1 Study design, setting, and recruitment of patients

This prospective exploratory pilot study was approved by the local ethical committee of the University of Tuebingen.

Between November 2010 and December 2012 we enrolled consecutive patients presenting with syncope at the medical emergency department of our tertiary center in Tuebingen, Germany. This collective was derived from a previous investigation of all-comers to the ED.^[15]^ According to the latest European Society of Cardiology guidelines syncope was defined as a transient loss of consciousness due to cerebral hypoperfusion characterized by rapid onset, short duration, and spontaneous complete recovery.^[20]^

**Figure 1:**
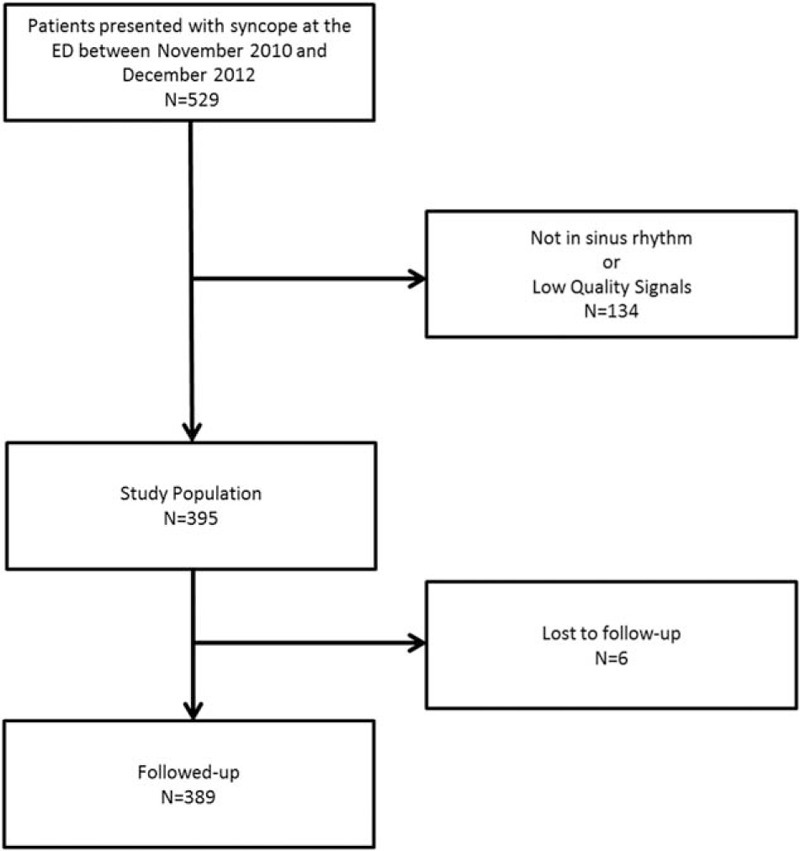
Patient recruitment**–**flow chart of patient selection.

The patient's ECG was recorded by routine monitoring devices (DASH 4000/5000 Teleguard General Electrics, Fairfield, CT). Monitoring was started directly after arrival at the ED. Treating physicians were blinded to the study design. Management or treatment was neither delayed nor changed due to study participation. Patients were included if they were in sinus rhythm (Fig. 1).

Baseline characteristics included sex, age, previous myocardial infarction, known congestive heart failure, arterial obstructive disease, a history of chronic renal insufficiency, and conventional cardiovascular risk parameters. Furthermore, the MEWS, the SFSS, and basic laboratory parameters were determined.

### 2.2 Assessment of DC

Technical details of the automated assessment of DC have been described elsewhere.^[21]^ The ECG recordings were checked for atrial fibrillation (AF) using a validated automated algorithm.^[22]^ Sections of AF were excluded. Both recordings with permanent atrial fibrillation and noisy low-quality signals were excluded from further analysis (Fig. 1).

Assessment of DC was performed by applying a signal processing algorithm called phase-rectified signal-averaging (PRSA), which is capable of extracting periodic components out of non-stationary, noisy signals.^[23]^ Briefly, DC calculation is performed in 5 steps: First, RR-intervals, which are longer than their predecessors, are identified and defined as anchors. Second, intervals surrounding the anchors, which may overlap, are defined. In the third and fourth steps, segments are aligned at the anchors and subsequently averaged. Fifth, the so-called PRSA-signal is quantified by Haar-wavelet analysis. The PRSA technology allows for several adjustments, which make the method more robust to artifacts and noise and improve agreement between automatically and manually processed ECGs.^[16]^ Here we used T = 4, (instead of 1; Eq. (2a) in^[23]^) and s = 5 (instead of 2; Eq. (8) in^[23]^).

Patients were stratified according to DC to following risk categories: high risk-DC <7 ms and low risk- DC ≥7 ms. The determination of this optimized cut-off value was performed according to “MaxSpSe” (Criterion based on simultaneously maximizing Sensitivity and Specificity).^[24,25]^

For using DC as a short-term measure, the number of anchors is more essential than the duration of ECG recordings. Results become most reliable if more than 150 anchors are identified. In the present study, the first 10 minutes of ECG recordings were used for the calculation of DC. In case of low-quality signals, observing time was extended up to 30 minutes until at least 200 anchors were identified.

### 2.3 MEWS

As previously described the MEWS is derived from respiratory- and heart rate, systolic blood pressure, body temperature, and level of consciousness. The score ranges from 0 (minimum risk) to 14 (maximum risk).^[26]^

### 2.4 SFSS

The SFSS defines patients at high risk, if they fulfill at least one of the following criteria: History of congestive heart failure, hematocrit <30%, new ECG changes, and shortness of breath or systolic blood pressure <90 mm Hg at presentation.^[9]^

### 2.5 Study endpoints

The primary endpoint was all-cause mortality 180 days after presentation to ED due to syncope.

### 2.6 Follow-up

Intrahospital deaths were recorded by the electronic information system. Patients were followed up either by presentation at our outpatient clinic or by telephone contact until 180 days after presentation to ED. Causes of death were classified into cardiac and noncardiac genesis by treating physicians not participating in the study.

### 2.7 Analysis

Continuous variables are presented as mean ± standard deviation and were compared using the Mann–Whitney *U* test. Qualitative data are presented both as absolute value and as percentages and were analyzed using the *χ*^2^ test. Receiver operating characteristic (ROC) curves were constructed for DC by plotting 1-specifity versus sensitivity. ROC curves were quantified by the area under the curve (AUC). The odds ratio (OR) of risk variables with the primary endpoint was calculated by univariable binary logistic regression analysis. Mortality rates were estimated by the Kaplan–Meier method.^[27]^ ORs are presented with 95% confidence intervals (CI). Differences were regarded as statistically significant, if the *P* value was less than .05. Statistical analyses were performed using SPSS 23.0. and CRAN R 3.3.0.

## 3 Results

### 3.1 Characteristics of study subjects

Five hundred twenty-nine patients presented to the ED due to syncope between November 2010 and December 2012. Three hundred ninety-five of these patients were enrolled in the study, whereas 134 were excluded due to either absence of sinus rhythm or noisy low-quality ECG signals (Fig. 1). Six patients (3.2%) were lost to follow-up. 50.9% of patients were women, mean age was 57.1 ± 20.0 years. One hundred fifty patients (38.0%) received outpatient treatment after evaluation by ED staff. Further baseline characteristics are presented in Table 1.

**Table 1:**
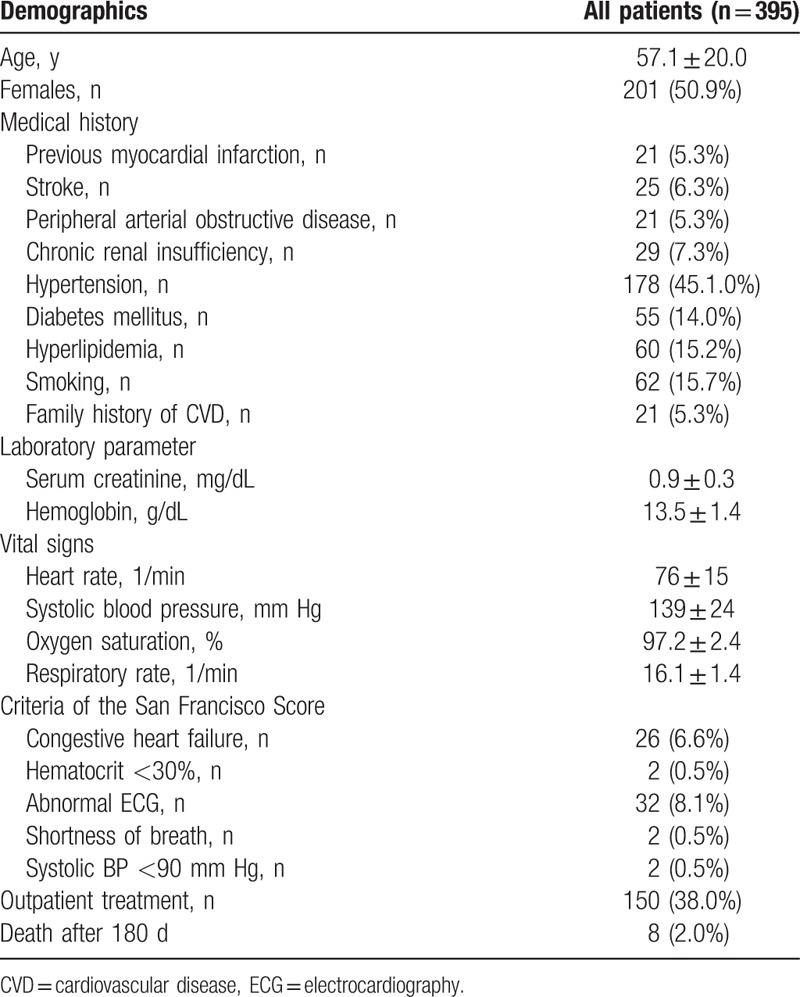
Baseline characteristics and outcomes of the study population.

### 3.2 Main results

All-cause mortality 180 days after presentation to ED was 2% (8 patients). 62.5% of them died due to a cardiac cause, 4 patients suffered a sudden cardiac death. The medical history of these patients is described in Table 2.

**Table 2:**
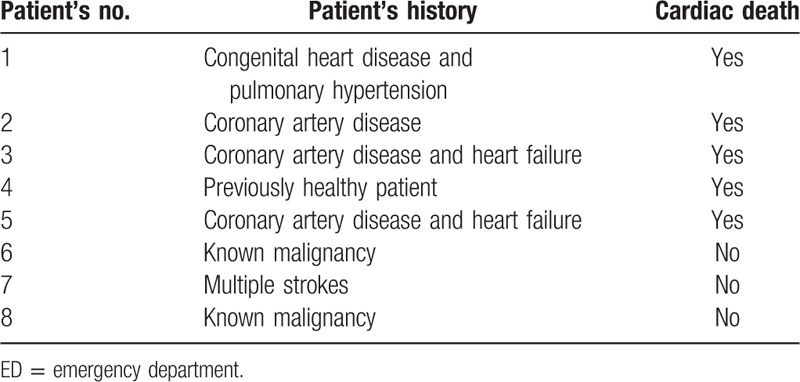
History of the patients who died while a 180-day period after admission to ED.

DC was significantly lower in the group of nonsurvivors as compared with survivors (3.1 ± 2.5 ms vs 6.7 ± 2.4 ms; *P* < .001) (Table 3). The SFSS failed at identifying 4 of 8 nonsurvivors (50%) as high risk patients whereas 13.7% of the survivors were stratified false-positive. However, the conventional risk score MEWS was comparable in both groups (2.1 ± 0.8 vs 2.1 ± 1.0; *P* = .84). Hemoglobin level at admission was significantly lower in the group of nonsurvivors (11.9 ± 2.5 g/dL vs 13.5 ± 1.4 g/dL; *P* = .03), whereas sex (37.5% vs 51.2%; *P* = .44), age (67.0 ± 19.9 vs 56.9 ± 20.0; *P* = .15), and serum creatinine level (1.3 ± 0.8 mg/dL vs 0.9 ± 0.3 mg/dL; *P* = .06) showed no significant difference. DC was significantly higher in the group of outpatients compared with patients who were admitted to hospital (7.1 ± 2.2 vs 6.3 ± 2.5 *P* < .001).

**Table 3:**
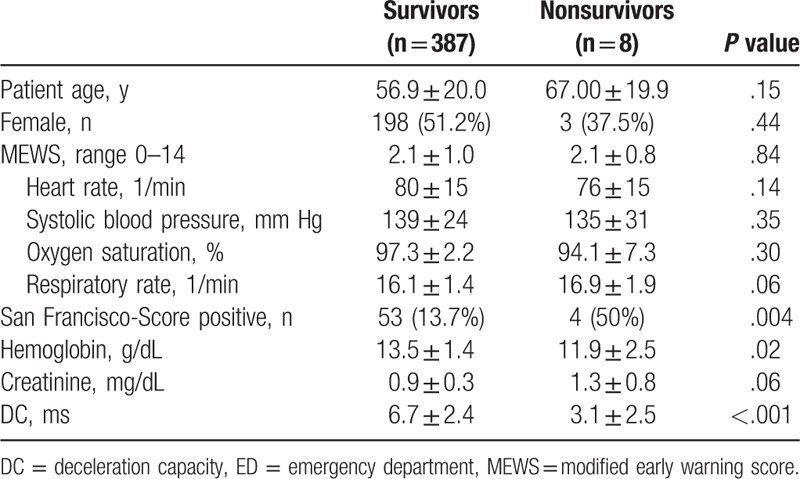
Characteristics of survivors and nonsurvivors 180 d after ED admission.

**Figure 2:**
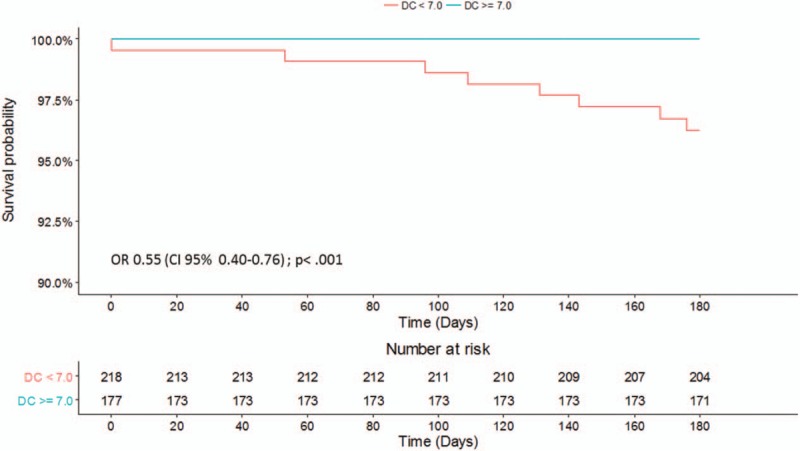
Kaplan–Meier survival curves—cumulative 180-day mortality of patients admitted to ED due to syncope stratified by deceleration capacity (DC). ED = emergency department.

One hundred seventy-seven (44.8%) of the 395 patients had a DC ≥7ms. No patient with a favorable DC died (0.0% vs 3.7%; *P* = .01) (Table 4, Fig. 2). Patients with a DC≥7 ms were significantly younger (48.45 ± 19.1 vs 64.13 ± 18.0; *P* < .001) and had lower serum creatinine levels (0.9 ± 0.2 vs 1.0 ± 0.3; *P* = .01) but were comparable regarding sex, hemoglobin level, and MEWS.

**Table 4:**
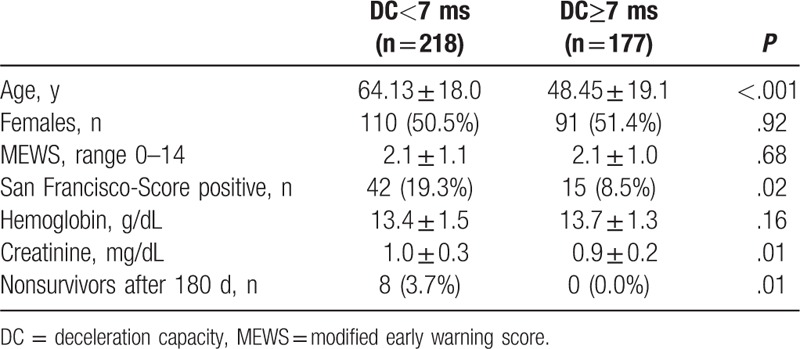
Comparison of baseline characteristics and mortality rate by DC status.

**Figure 3:**
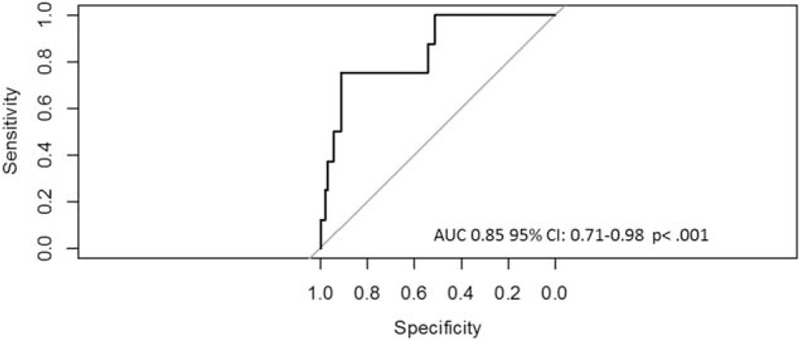
ROC curve of DC prediction model—receiver operating curve of DC for prediction of 180 day-mortality after admission to ED due to syncope. DC = deceleration capacity, ED = emergency department, ROC = receiver operating characteristic.

DC, the SFSS and hemoglobin levels were significant predictors for the primary endpoint yielding an OR of 0.55 (95% CI 0.40–0.76, *P* < .001), of 6.30 (95% CI 1.53–25.96, *P* = .01) and of 0.46 (95% CI 0.28–0.74, *P* = .001), respectively. Figure 3 shows the ROC curve for the prediction of all-cause mortality 180 days after syncope. DC yielded an AUC of 0.85 (95% CI 0.71–0.98 *P* < .001).

## 4 Discussion

This study shows that the DC of heart rate provides important prognostic information regarding 180 days-mortality in patients presenting to the ED with syncope. In our cohort, no patient with a favorable DC died while follow-up. Conventional risk predictors like the MEWS did not predict mortality in our cohort. The SFSS failed at identifying 4 of the 8 non-survivors as high risk. DC was significantly higher in the group of survivors, was associated with 180-days-mortality, and showed excellent sensitivity and specificity, as shown by the ROC curve.

The identification of high-risk patients with syncope is challenging. Previous studies were performed to develop eligible scores, but none of them were implemented into daily patient care. Both the Osservatorio Epidemiologico sulla Sincope nel Lazio (OESIL-Score) and the Evaluation of Guidelines in SYncope Study score identify patients at high risk by screening them for cardiac symptoms or pathological ECGs.^[8,10]^ However, noncardiac threatening conditions leading to loss of consciousness may not be covered by these risk stratification tools. BNP, hemoglobin, heart rhythm monitoring, ECG, clinical examination, and vital signs are required to calculate the risk stratification of syncope in the emergency department score.^[11]^ High-risk patients with a DC < 7 may benefit from these comprehensive tests, however, in times of ED overcrowding prompt identification of patients at low risk is needed to prevent resource-wasting.

Hence, markers that allow for a precise real-time risk prediction in syncope patients are warranted. Our results demonstrate that DC is a powerful and easy applicable tool to identify patients at low risk. These patients might be discharged both safely and promptly. Further acute diagnostics might not be required in the ED setting and can be performed by elective outpatient appointments later on. Remarkably, almost 45% of our patient collective was marked as low-risk by a favorable DC, independent of the underlying condition. These patients could have been discharged promptly. In this manner, both economical and personal resources can be used for critical ill patients instead.

Risk stratification by DC is done efficiently and effectively. The calculation of DC can be performed by easily manageable software out of standard heart rhythm monitoring recordings. Risk prediction by DC is independent of the investigator, noninvasive, nonexpensive, and is able to preserve resources.

The pathophysiological mechanisms of critical syncope patients leading to an impaired DC are not investigated in detail. Most likely, the development of an unfavorable DC in this collective is similar to those with heart failure or acute myocardial infarction.^[18,28]^ Neurohumeral adaptions like the activation of both the sympathetic nerve and the renin–angiotensin–aldosterone system induce an inability of the ANS to react to vagal influences.^[29]^ DC is an integral measure of the ability to oscillate the heart rate and can quantify this imbalance. Hence, this marker might be such a strong risk predictor.

Remarkably, hemoglobin level was also shown to be a risk factor for syncope patients. This result is in line with previous studies.^[9,11]^ Low hemoglobin level might identify patients suffering from syncope due to any bleeding. However, it might not be eligible as a risk prediction tool due to its inability to identify threatening cardiac syncope.

The limitations of our study need to be mentioned. First, DC cannot be calculated in patients with atrial fibrillation. In these cases risk stratification needs to be performed by conventional scores. Second, our study was purely explorative, observational and hypothesis-generating, further prospective investigations are needed to confirm if the application of DC as a risk predictor in syncope patients leads to a better outcome. Third, 6 patients were lost to follow-up which attenuates the value of our results. Fourth, due to the small amount of endpoints multivariable analysis and comparison to previous scores was not eligible. Further studies should clarify potential superiority of DC.

DC of heart rate is a strong predictor of 180 days mortality in syncope patients presenting to ED. The application of this new tool, which can be easily integrated in several monitoring systems, enables the ED physician to identify both critical cases and patients at low-risk qualifying for early discharge.
